# Disruptions in hepatic glucose metabolism are involved in the diminished efficacy after chronic treatment with glucokinase activator

**DOI:** 10.1371/journal.pone.0265761

**Published:** 2022-03-21

**Authors:** Yoshinori Tsumura, Yu Tsushima, Azusa Tamura, Hirotsugu Kato, Tsunefumi Kobayashi

**Affiliations:** Pharmacology Research Department, Teijin Institute for Bio-medical Research, Teijin Pharma Limited, Hino, Tokyo, Japan; Max Delbruck Centrum fur Molekulare Medizin Berlin Buch, GERMANY

## Abstract

Glucokinase activators are regarded as potent candidates for diabetes treatment, however, in clinical studies on patients with type 2 diabetes, a diminishing efficacy was observed after chronic treatment with them. The mechanism of this reduction has not been elucidated, and whether it is a class effect of glucokinase activators remains inconclusive. Here, we firstly identified a diabetic animal model that shows the diminished efficacy after long-term treatment with MK-0941, a glucokinase activator that exhibited diminished efficacy in a clinical study, and we analyzed the mechanism underlying its diminished efficacy. In addition, we evaluated the long-term efficacy of another glucokinase activator, TMG-123. Goto-Kakizaki rats were treated with MK-0941 and TMG-123 for 24 weeks. The results showed that glycated hemoglobin A1C levels and plasma glucose levels decreased transiently but increased over time with the continuation of treatment in the MK-0941-treated group, while decreased continuously in the TMG-123-treated group. Only in the TMG-123-treated group, higher plasma insulin levels were shown at the later stage of the treatment period. For the mechanism analysis, we conducted a hepatic enzyme assay and liver perfusion study in Goto-Kakizaki rats after chronic treatment with MK-0941 and TMG-123, and revealed that, only in the MK-0941-treated group, the activity of glucose-6-phosphatase was increased, and hepatic glucose utilization was decreased compared to the non-treated group. These data indicate that disruptions in hepatic glucose metabolism are involved in the diminished efficacy of glucokinase activators.

## Introduction

Type 2 diabetes is a metabolic disease characterized by chronic hyperglycemia, and the number of patients experiencing this disease is projected to continue increasing globally [1]. The recommended treatment for type 2 diabetes includes exercise, diet, and drug therapy [2]. Although various classes of therapeutic agents are currently utilized for the treatment of diabetes, patients who achieve treatment goals are limited and there remains a continued need for novel drugs that facilitate such achievement.

Glucokinase (GK) activators are regarded as potent candidates for antidiabetic drugs, exhibiting a novel mechanism of action. GK is mainly expressed in the liver and pancreas, and catalyzes the first reaction in glucose metabolism. The affinity of GK for glucose is around the physiological blood glucose levels, thereby regulating insulin secretion as well as the rate of hepatic glucose uptake in response to rising blood glucose levels [3–5]. These characteristics allow GK to act as a glucose sensor and to regulate whole-body glucose homeostasis. Therefore, activation of GK is expected to be a successful therapeutic strategy for type 2 diabetes [6]. Previous clinical studies have demonstrated the preferable efficacy of GK activators in patients with type 2 diabetes, however, the efficacy of some GK activators diminished after 8–12 weeks or longer treatment [7–9]. The mechanism of the diminished efficacy of GK activator treatment has not been elucidated, and whether such diminishing effect is a class effect remains inconclusive. Diminished efficacy is a crucial problem from the perspective of drug development of GK activators as anti-diabetic agents, therefore, it is important to elucidate the mechanism and develop novel candidates that can avoid this reduction.

In the present study, we firstly identified an animal model that shows the diminished efficacy after chronic treatment with MK-0941, a glucokinase activator that exhibited diminished efficacy in a clinical study, and we analyzed the mechanism of the diminished efficacy in this model. At the same time, we assessed the maintenance of efficacy of another glucokinase activator, TMG-123, which is previously demonstrated its potent and durable antidiabetic effects in animal models of type 2 diabetes [10].

## Material and methods

### Animals

Male Goto-Kakizaki rats and Wistar rats were obtained from Japan SLC, Inc. (Shizuoka, Japan). All animals were housed under a 12 h light-dark cycle and were fed ad libitum a CE-2 (CLEA Japan, Inc., Tokyo, Japan), All experimental procedures were approved by the Animal Care and Use Committee of Teijin Institute for Bio-Medical Research. All efforts were made to minimize suffering. All surgeries were performed under anesthesia by intraperitoneal injection of 50 mg/kg sodium pentobarbital (Somnopentyl; Kyoritsu Seiyaku Corporation, Tokyo, Japan). These laboratory animal facilities were accredited by the Center for Accreditation of Laboratory Animal Care and Use, Japan Health Sciences Foundation (Certification Number: 13–066, 15–051, 16–066).

### Chronic treatment study

**Fig 1:**
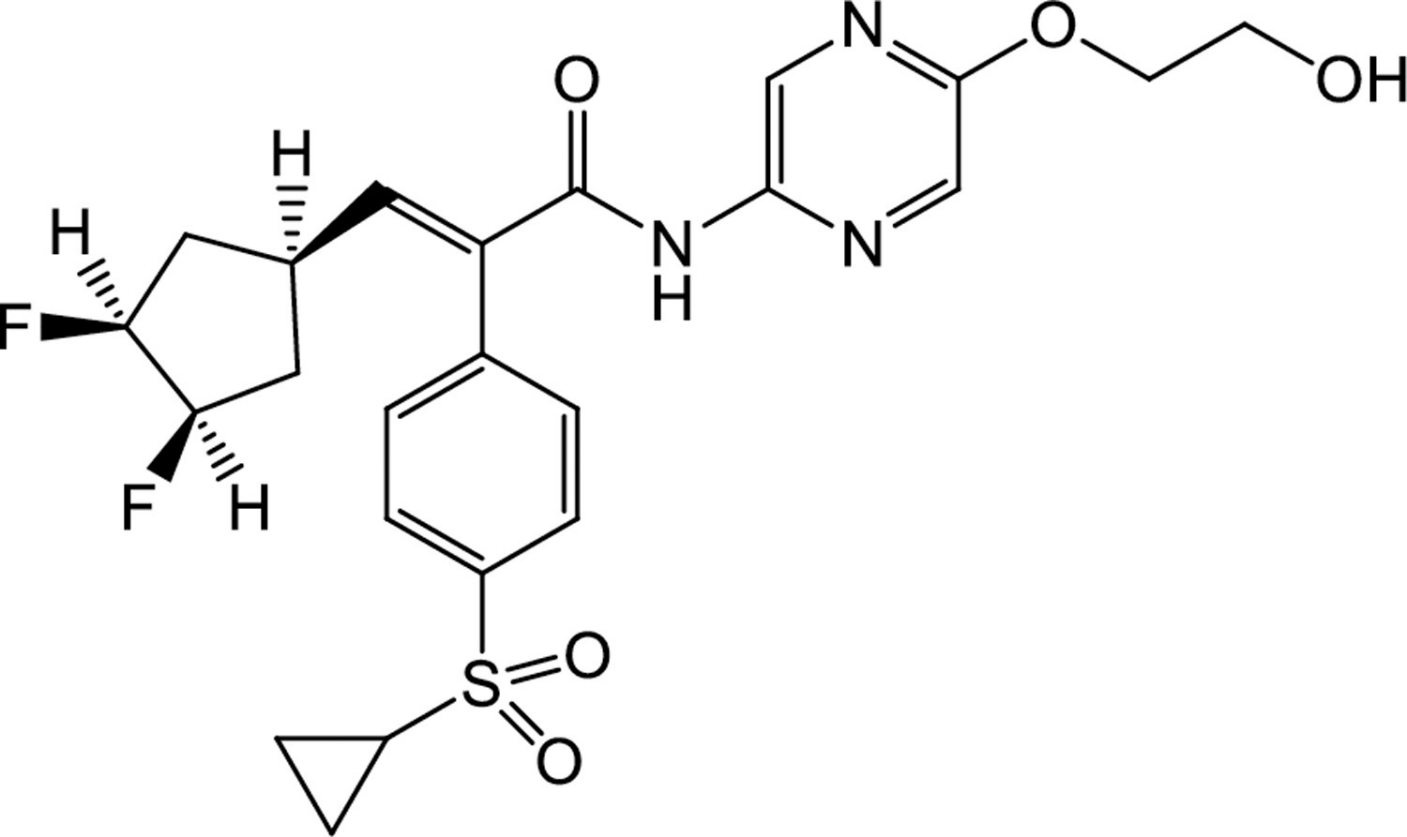
Chemical structure of the glucokinase activator TMG-123.

Goto-Kakizaki rats at 7 weeks of age were fed for a maximum of 24 weeks with CE-2, CE-2 containing 0.1% MK-0941 (chemical structure shown in the previous report [11], compound synthesized in NARD CHEMICALS, LTD. (Osaka, Japan) and pellets made in CLEA Japan, Inc.), or powder CE-2 containing 0.2% TMG-123 (chemical structure shown in Fig 1, compound synthesized and powder mixed with rocking mill in Teijin Pharma Limited. (Tokyo, Japan)), and Wistar rats at 7 weeks of age were fed for a maximum of 24 weeks with CE-2. Blood was sampled for the measurement of hemoglobin A1C (HbA1c) levels (measured at 9:00 only), plasma glucose levels (at 9:00 before treatment and at 9:00 and 21:00 in the treatment period), and plasma insulin levels (at 9:00 before treatment and at 9:00 and 21:00 in the treatment period) every 4 weeks. HbA1c levels were measured by DCA Vantage Analyzer (Siemens Japan, Tokyo, Japan), plasma glucose was measured by Glucose CII-Test Wako (FUJIFILM Wako Pure Chemical Corporation, Osaka, Japan), and plasma insulin was measured by High-sensitive Measurement Kit for Insulin (Morinaga Institute of Biological Science, Inc., Kanagawa, Japan).

### Liver perfusion study

At 20 weeks in the chronic treatment study, hepatic glucose utilization, uptake, and production were evaluated by the liver perfusion study with uniformly ^13^C-labeled glucose ([U-^13^C]-glucose) in accordance with the previous report [12]. Rats were anesthetized and the peritoneal cavity was opened. Thirty units of heparin Na (Nipro Corporation, Osaka, Japan) was injected into the inferior vena cava, and simultaneously the blood was sampled for the measurement of plasma glucagon by Glucagon ELISA kit (Mercodia, Uppsala, Sweden). After heparin injection, the left branch of the portal vein was ligated. Subsequently, the portal vein and the infrahepatic inferior vena cava were cannulated. Rats were euthanized by exsanguination with perfusion of 5 mmol/L D-glucose in Krebs-Henseleit bicarbonate buffer (KHBB) (118.5 mmol/L NaCl, 4.7 mmol/L KCl, 2.5 mmol/L CaCl_2_, 1.2 mmol/L KH_2_PO_4_, 1.2 mmol/L MgSO_4_, 24.9 mmol/L NaHCO_3_, 33°C) bubbled with 95% O_2_/5% CO_2_ via the portal vein under anesthetization. After confirmation of death, the suprahepatic inferior vena cava was ligated and the right lateral lobe of the liver was perfused via the portal vein at a flow rate of 6 mL/min (3 mL/min/g liver) for 30 minutes as the stabilization period with 5 mmol/L D-glucose in KHBB bubbled with 95% O_2_/5% CO_2_. Following, the perfusate was switched to 5 mmol/L [U-^13^C]-glucose in KHBB bubbled with 95% O_2_/5% CO_2_. The effluent perfusates were collected as measurement samples from the inferior vena cava. The glucose concentrations of collected samples were measured by a Glucose CII-Test Wako. Glucose utilization was calculated from the difference in the glucose concentration between baseline perfusate and effluent perfusate. After measuring the abundance ratio of [U-^12^C]-glucose and [U-^13^C]-glucose in these perfusates by Gas Chromatography/Mass Spectrometry, glucose uptake was determined as the decrease of [U-^13^C]-glucose concentration and glucose production was determined as the increase of [U-^12^C]-glucose concentration from baseline perfusate to effluent perfusate.

### Hepatic enzyme assay

At 20 weeks in the chronic treatment study, rats were sacrificed under anesthesia in the liver perfusion study, and liver was collected and immediately frozen with liquid nitrogen. The liver was homogenized at 4°C in Takara Biomasher Standard (Takara Bio INC, Shiga, Japan) containing homogenization buffer (50 mmol/L HEPES, 5 mmol/L MgCl_2_, 100 mmol/L KCl, 1 mmol/L EDTA, 1% triton-X, Protease inhibitor cocktail (Sigma-Aldrich, MO, USA)). The lysate was centrifuged with 12000 g and supernatant was collected for GK enzyme assay. The assay method for hepatic GK activity essentially described by Desai [13]. GK activity was estimated as the difference in phosphorylation activity when samples assayed at 100 mmol/L and 0.5 mmol/L glucose. For the Glucose-6-phosphatase (G6Pase) assay, the supernatant was centrifuged 105000 g and decant lysate were diluted with Cacodylic acid buffer (50 mmol/L Cacodylic acid-HCl, 2 mmol/L EDTA, pH6.5). The assay method for G6Pase activity essentially described by Burchell [14]. The samples were incubated with 150 mmol/L Glucose-6-phosphate solution for 10 minutes at 30°C and G6Pase activity was estimated as the amount of phosphate after incubation.

### Statistical analysis

Statistical analysis was performed using SAS software version 9.2 (SAS Institute Inc., Cary, NC, USA). Data are expressed as mean + SEM or mean ± SEM in each figure. The statistical significance of differences was assessed using two-tailed Student’s t-test and the Aspin-Welch test for single comparisons. Differences were considered significant when p values were < 0.05 in all tests.

## Results

### Chronic treatment study of GK activators in Goto-Kakizaki rats

**Fig 2:**
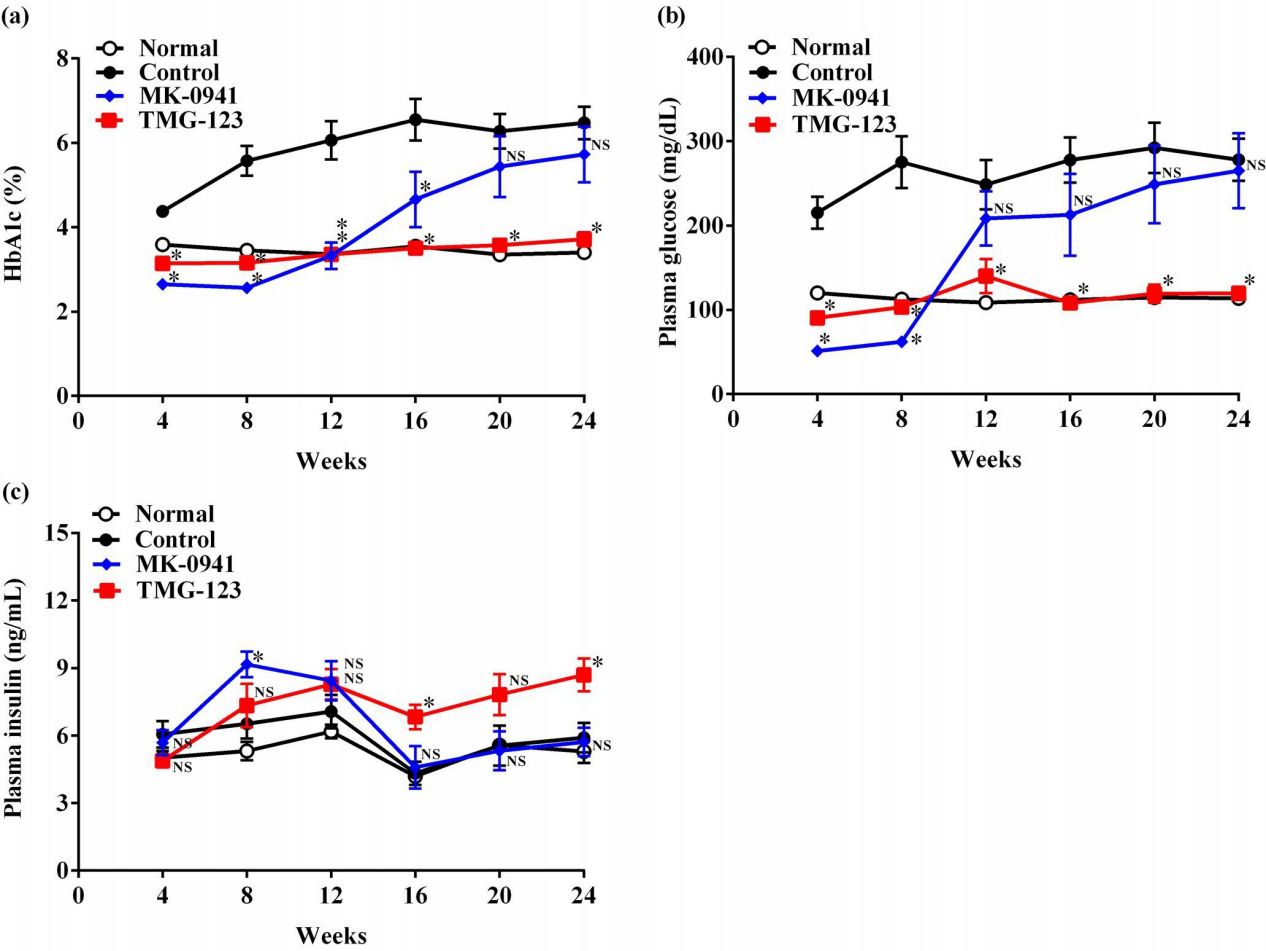
HbA1c, plasma glucose and plasma insulin levels in the chronic treatment study. Time course of (a) HbA1c level, (b) plasma glucose level, and (c) plasma insulin level during the chronic treatment study. Normal; Wistar rats, Control; Goto-Kakizaki rats fed normal diet, n = 7–8. *p < 0.05, NS = not significant.

First, we treated Goto-Kakizaki rats with MK-0941 and TMG-123 for 24 weeks (Fig 2). At baseline, there were no significant differences between the Control, MK-0941, and TMG-123 groups in HbA1c, plasma glucose, and plasma insulin levels (S1 Fig). Four weeks after the beginning of treatment, HbA1c levels of both MK-0941 and TMG-123 groups decreased in comparison with the Control group and reached lower levels than the Normal group (Fig 2A). However, with the continuation of treatment, HbA1c levels gradually increased in the MK-0941 group, and at 24 weeks, the MK-0941 group and the Control group did not show significantly different HbA1c levels. Conversely, HbA1c levels did not change substantially throughout the treatment period in the TMG-123 group, and at 24 weeks, the TMG-123 group showed significantly lower HbA1c levels compared to the Control group. Similarly, 4 weeks after the beginning of treatment, both MK-0941 and TMG-123 groups exhibited plasma glucose-lowering effects compared to the Control group, and plasma glucose levels of both groups reached lower levels than the Normal group (Fig 2B). However, with the continuation of treatment, plasma glucose levels markedly increased from 8 to 12 weeks in the MK-0941 group, and at 24 weeks of treatment, MK-0941 and Control groups did not show significantly different plasma glucose levels. Conversely, plasma glucose levels did not change considerably throughout the treatment period in the TMG-123 group, and at 24 weeks, the TMG-123 group showed significantly lower plasma glucose levels compared to the Control group. Based on the results of the MK-0941 group, the diminished efficacy after chronic treatment with MK-0941 was shown in Goto-Kakizaki rats. As TMG-123 sustained its efficacy for 24 weeks in this animal model, a difference in the durability of efficacy between the two GK activators was demonstrated.

In this study, plasma insulin levels in the MK-0941 group were significantly higher or tended to be higher than those in the Control group at 8 and 12 weeks, but similar to those in the Control group after 16 weeks (Fig 2C). Plasma insulin levels in the TMG-123 group were significantly higher or tended to be higher than those in the Control group after 8 weeks.

### Mechanism analysis of diminished efficacy

**Fig 3:**
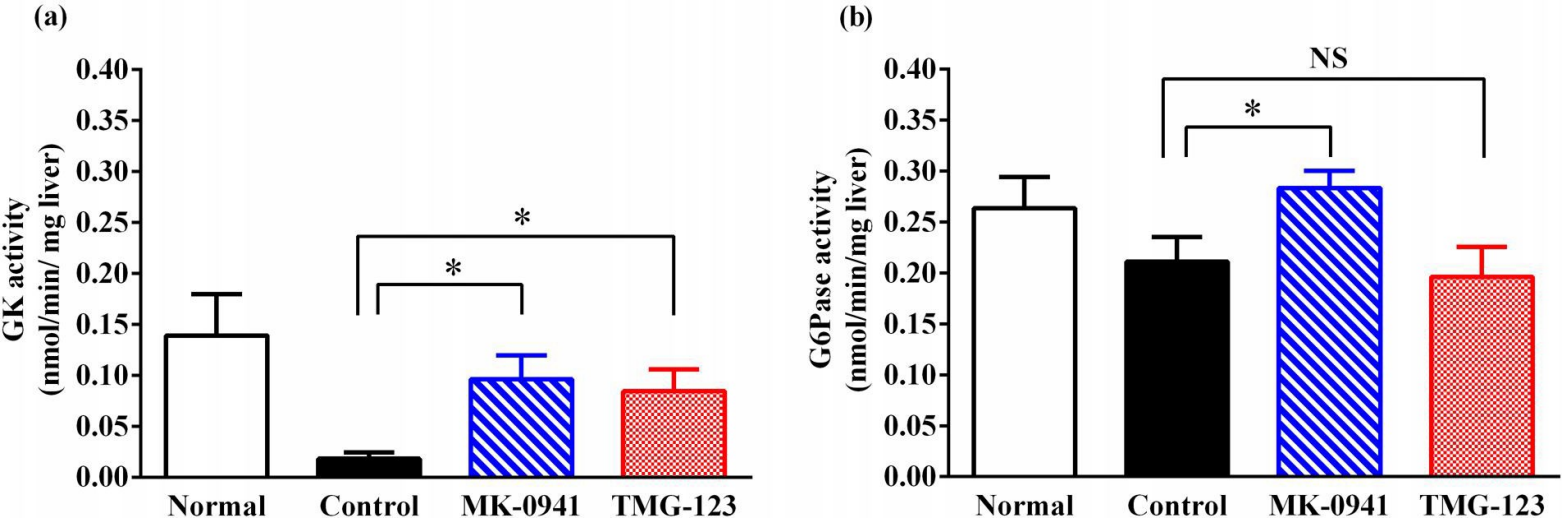
GK and G6Pase activity in the liver after 20-week treatment with GK activators. (a) GK activity and (b) G6Pase activity at week 20 of the chronic treatment study. Normal; Wistar rats, Control; Goto-Kakizaki rats fed normal diet, n = 7–8. *p < 0.05, NS = not significant.

Next, we assessed the hepatic enzyme activity of GK and G6Pase, a rate-limiting enzyme of gluconeogenesis that counteracts GK, in Goto-Kakizaki rats after chronic treatment with GK activators. Hepatic GK activities in the MK-0941 and TMG-123 groups were significantly greater than those in the Control group at 20 weeks of treatment (Fig 3A). Conversely, compared to the Control group, the MK-0941 group also had significantly greater hepatic G6Pase activity, whereas the TMG-123 group showed similar activity (Fig 3B). These results indicate that increased hepatic G6Pase activity contributes to diminished efficacy after chronic MK-0941 treatment.

**Fig 4:**
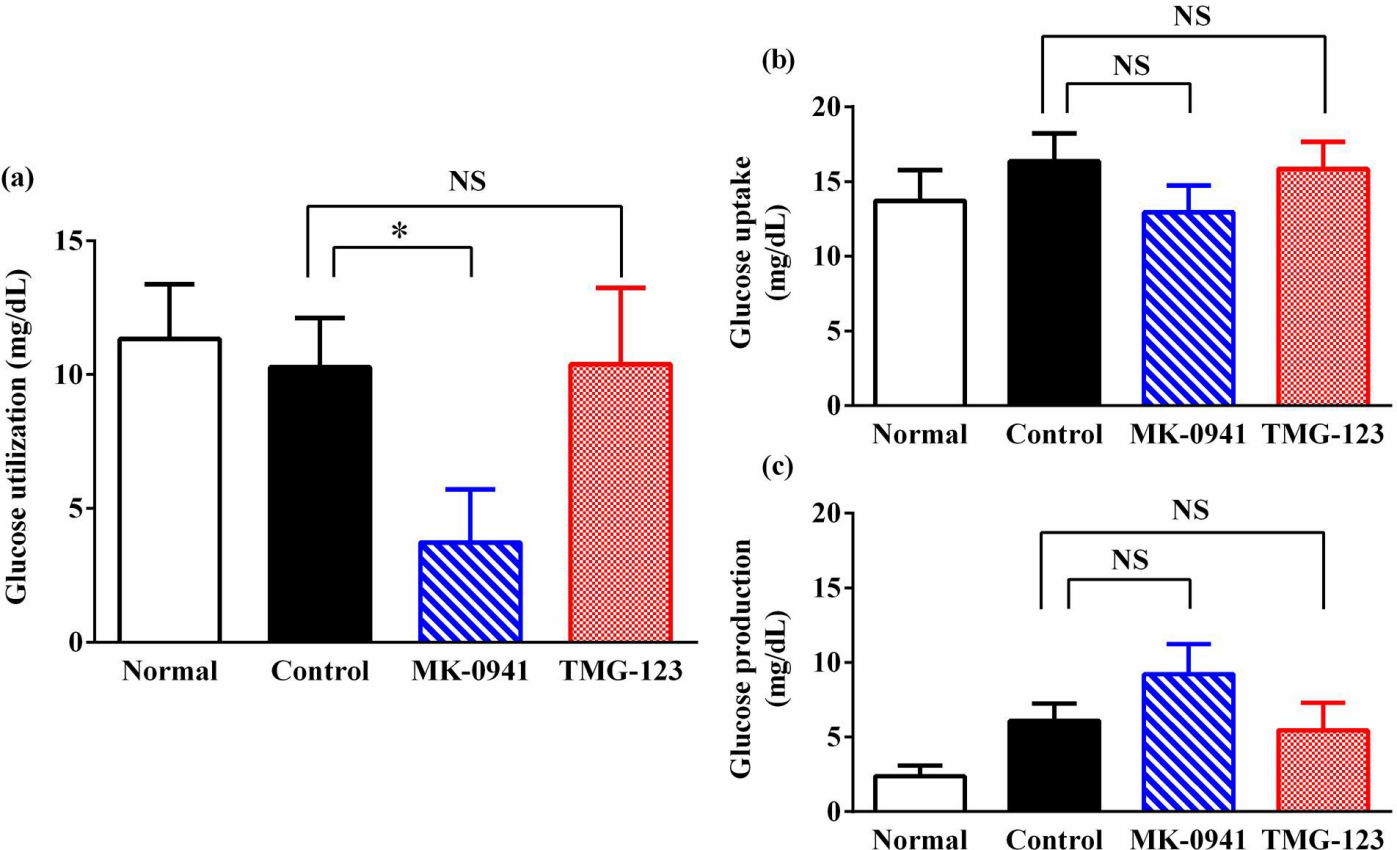
Glucose utilization, uptake, and production after 20-week treatment with GK activators. (a) Glucose utilization, (b) glucose uptake, and (c) glucose production was evaluated with the liver perfusion study at week 20 in the chronic treatment. Normal; Wistar rats, Control; Goto-Kakizaki rats fed normal diet, n = 5–7. *p < 0.05, NS = not significant.

GK activators have been reported to exert glucose-lowering effects by enhancing hepatic glucose utilization [10, 11]. To further investigate hepatic glucose metabolism after chronic treatment with GK activators, we conducted a liver perfusion study and assessed hepatic glucose utilization. As a result, the MK-0941 group exhibited significantly lower glucose utilization than the Control group (Fig 4A). In contrast, glucose utilization in the TMG-123 group was similar to that in the Control group. These results suggest that chronic treatment with MK-0941 causes a decrease in hepatic glucose utilization, which is involved in its diminished efficacy.

Hepatic glucose utilization was determined as the difference between the hepatic glucose uptake and production [12]. We analyze the hepatic glucose uptake and production by perfusing [U-^13^C]-glucose. As a result, the MK-0941 group tended to exhibit lower glucose uptake and higher glucose production compared to the Control group (Fig 4B and 4C). Conversely, glucose uptake and production in the TMG-123 group were similar to those in the Control group (Fig 4B and 4C). These results indicate that decreased hepatic utilization in the MK-0941 group after chronic treatment is attributed to both hepatic glucose uptake decrease and hepatic glucose production increase.

## Discussion

A clinical trial of MK-0941 in patients with type 2 diabetes showed that blood glucose control significantly improved at week 14, but this improvement did not continue until week 30 [7]. The average HbA1c level of the patients enrolled in this study was around 9%, showing that the average plasma glucose levels were around 240 mg/dL [15]. It is worth noting that the patients received insulin therapy, indicating that they exerted both insulin resistance and decreased insulin secretion. In the present study, we examined the maintenance of plasma glucose-lowering effects with a 24-week MK-0941 treatment in Goto-Kakizaki rats, a diabetes model whose average plasma glucose levels were similar to the patients in the clinical study, around 240 mg/dL (Fig 2B), and exhibited both insulin resistance and decreased insulin secretion [16, 17]. Results showed that both HbA1c and plasma glucose levels decreased from 4 weeks to 8 weeks of treatment, which reached lower levels than the Normal group, but began to increase thereafter and were similar to the Control group at 24 weeks. These results demonstrated that the diminished efficacy of MK-0941 observed in the clinical study was shown in a diabetic animal model. There have been no models reported that show the diminishing efficacy after long-term treatment with GK activators, therefore, this model is useful for the mechanistic analysis of diminished efficacy. In this model, TMG-123 continuously decreased HbA1c and plasma glucose levels until 24 weeks. These findings indicate that the diminished efficacy of GK activators after long-term administration is not common for all compounds.

To analyze the mechanism of the diminished efficacy of MK-0941, we conducted a hepatic enzyme activity assay, focusing on hepatic GK protein itself and hepatic G6Pase, which counteracts GK. Hepatic GK activities in the MK-0941 and TMG-123 groups were greater than those in the Control group. Conversely, hepatic G6Pase activity in the MK-0941 group was greater than that in the Control group, whereas it was similar in the TMG-123 group. Although it is difficult to discuss quantitatively the results of these enzyme assays because they are independent assays, GK activity of TMG-123 group was relatively dominant to G6Pase activity compared to Control and MK-0941 group; the differences between GK and G6Pase activity were similar in Control and MK-0941 groups, approximately 0.18 nmol/min/mg liver higher in G6Pase assay, while being relatively small in the TMG-123 group, approximately 0.10 nmol/min/mg liver higher in G6Pase assay (Fig 3). Although hepatic GK activities increased in both MK-0941 and TMG-123, G6Pase activity increase in the MK-0941 group may lead to different results of diminished efficacy. Previous studies showed that treatment with glucokinase activators induced hepatic gene expression of G6Pase (G6pc) in hepatocytes and animal models [18, 19], which is consistent with our results in the MK-0941 group. Since these studies indicated that glucose metabolites and carbohydrate response element binding protein are involved in the increase in G6pc expression, it is possible that the glucose metabolites increased or transcription factors activated by chronic treatment with MK-0941 exerted a feedback mechanism, resulting in an induction of G6Pase expression and diminished efficacy.

Hepatic perfusion experiments in Goto-Kakizaki rats treated with GK activators for 20 weeks showed that glucose utilization was markedly decreased in the MK-0941 group compared to the Control group. In this hepatic perfusion experiment, glucose uptake and glucose production were estimated using ^13^C-labeled glucose as a tracer. Results showed that glucose uptake tended to decrease, and glucose production tended to increase in the MK-0941 group. Based on these findings, it was postulated that chronic treatment with MK-0941 induces an imbalance between glucose uptake and glucose production, which results in a decrease in the hepatic glucose utilization, thereby leading to an attenuation of the plasma glucose-lowering effect. Conversely, hepatic glucose utilization in the TMG-123 group was similar to that in the Control group, indicating that disruption of hepatic glucose metabolism did not occur after chronic treatment with TMG-123. In the perfusion study, ^13^C-labeled glucose is taken up in the liver and converted to ^13^C-labeled glucose-6-phosphate by GK, and ^13^C-labeled glucose-6-phosphate is converted to ^13^C-labeled glucose by G6Pase, which is then released into the effluent perfusate. Therefore, substantial glucose uptake decreases when G6Pase activity is high. Although the result of high GK activity in the MK-0941 group appears to be inconsistent with the results of the decreased hepatic glucose uptake in the MK-0941 group, relatively high G6Pase activity in the MK-0941 group may result in low substantial glucose uptake and high glucose production.

GK activators are reported to enhance insulin secretion and decrease blood glucose levels [6]. A previous report showed that mice with an activating genetic modification of GK in the pancreatic β cells transiently exhibit a marked decrease in blood glucose, however, this effect does not continue as pancreatic β cell apoptosis occurs due to glucotoxicity, leading to an increase in blood glucose to a level similar to that of normal mice [20]. It has also been reported in a primary rat islet cell culture system that the induction of GK activators under hyperglycemic conditions triggers pancreatic β cell apoptosis [21]. Based on these findings, it has been assumed that the diminished efficacy was caused by the attenuation of insulin secretion by MK-0941 or the impairment of pancreatic β cells. In the present study, plasma glucose levels in the MK-0941 group markedly increased from 8 to 12 weeks (Fig 2B), and the diminished efficacy was presumed to occur during this period. Thus, we focused on whether the trend in plasma insulin levels changed during the same period in the same animals. At both 8 and 12 weeks, the plasma insulin level was greater in the MK-0941 group than in the Control group (Fig 2C), indicating that a trend in plasma insulin levels was not changed. These results indicate that the reduction of insulin secretion by MK-0941 as well as pancreatic β cell apoptosis is not the main reason for the diminished efficacy of MK-0941 in Goto-Kakizaki rats.

In this study, chronic treatment with MK-0941 and TMG-123 resulted in different outcomes in terms of the maintenance of efficacy. While the slight difference in the glucose levels at 4–8 weeks between the TMG-123 and the MK-0941 group might have caused these different outcomes (Fig 2B), the glucose levels of both groups were lower than those of the Normal group, indicating that the duration of efficacy of the two GK activators was assessed under the condition that the efficacy was sufficient for the two GK activators to act as antidiabetic drugs. The levels of plasma glucagon, known to be secreted in response to hypoglycemia and stimulate gluconeogenesis, in both the MK-0941 and TMG-123 groups were not elevated compared with those in the Control group at week 20 of the chronic treatment (S2 Fig), suggesting that they do not have pharmacological effects on glucagon secretion. Furthermore, the plasma glucose levels in the MK-0941 group increased above the hypoglycemic range and reached levels similar to those in the Control group at a later stage of the treatment period (Fig 2B). Although plasma glucagon levels could not be evaluated throughout the treatment period, these data suggest that glucagon secretion as a pharmacological effect of GK activators or metabolic adaptations may not be involved in the diminished efficacy. Regarding different outcomes in duration of efficacy, we considered two possible reasons from the perspective of sustained efficacy of TMG-123. First, TMG-123 did not cause the disruption of hepatic glucose metabolism. Previous studies indicated that the treatment with GK activators leads to numerous changes in hepatic glucose metabolism [22] and the effects of GK activators on GK protein conformation, GK-glucokinase regulatory protein interaction, and glycogen phosphorylase are different based on their chemical structure [23, 24], suggesting that the effects on hepatic glucose metabolism differ for each GK activator. To elucidate the precise mechanism of the diminished efficacy of GK activators, further investigation focused on the differences in the effects on glucose metabolism between MK-0941 and TMG-123 will be required. Second, plasma insulin levels of the TMG-123 group were increased at a later stage of the treatment period (Fig 2C). In our previous report, TMG-123 was shown to exert a glucose-lowering effect without affecting insulin levels in the diabetic animal models including Goto-Kakizaki rats [10]. This is consistent with the results of this study indicating that plasma glucose levels decreased without an increase in plasma insulin levels in the TMG-123 group at week 4 (Fig 2B and 2C). Therefore, the increase of plasma insulin levels at a later stage of the treatment period may be occurred due to the restoration of the function of pancreatic β cells which were caused by the protection from glucotoxicity rather than the effect of TMG-123 on the pancreatic β cells. This restoration might have contributed to the further glucose-lowering effect of TMG-123 at the later stage of the treatment period. Recently, other antidiabetic agents have been reported to improve pancreatic β cell function in patients with type 2 diabetes by lowering the plasma glucose concentration [25, 26], suggesting that the reduction in plasma glucose levels by therapeutic intervention results in the improvement of the β cell function in type 2 diabetes. Thus, GK activators that sustain glucose-lowering effects can also be expected to improve the function of pancreatic β cells in patients with type 2 diabetes.

In this study, we firstly identified the diabetic animal model that shows the diminished efficacy by long-term treatment with GK activator and found that disruptions in hepatic glucose metabolism may be involved in the diminished efficacy of GK activators. Glucokinases that do not exhibit disruptions in hepatic glucose metabolism and maintain higher insulin levels, such as TMG-123, are expected to sustain their efficacy after long-term treatment.

## Supporting information

S1 Fig HbA1c level, plasma glucose, and insulin levels before treatment with GK activators. (a) HbA1c level, (b) plasma glucose level, and (c) plasma insulin levels before treatment with GK activators (at baseline) in the chronic treatment study. Normal; Wistar rats, Control; Goto-Kakizaki rats fed normal diet in the treatment period, n = 7–8. NS = not significant.
(DOCX)

S2 Fig Plasma glucagon levels at 20-week treatment with GK activators. Plasma glucagon levels at week 20 of the chronic treatment study. Normal; Wistar rats, Control; Goto-Kakizaki rats fed normal diet, n = 7–8. NS = not significant.
(DOCX)
