# Supplementary material for: Disruptions in hepatic glucose metabolism are involved in the diminished efficacy after chronic treatment with glucokinase activator
Source: PLoS One. 2022 Mar 21;17(3):e0265761. doi: 10.1371/journal.pone.0265761 (PMC8936481; doi:10.1371/journal.pone.0265761)
Supplement: S1 Fig — (a) HbA1c level, (b) plasma glucose level, and (c) plasma insulin levels before treatment with GK activators (at baseline) in the chronic treatment study. Normal; Wistar rats, Control; Goto-Kakizaki rats fed normal diet in the treatment period, n = 7–8. NS = not significant. (DOCX) [file pone.0265761.s001.docx]

**
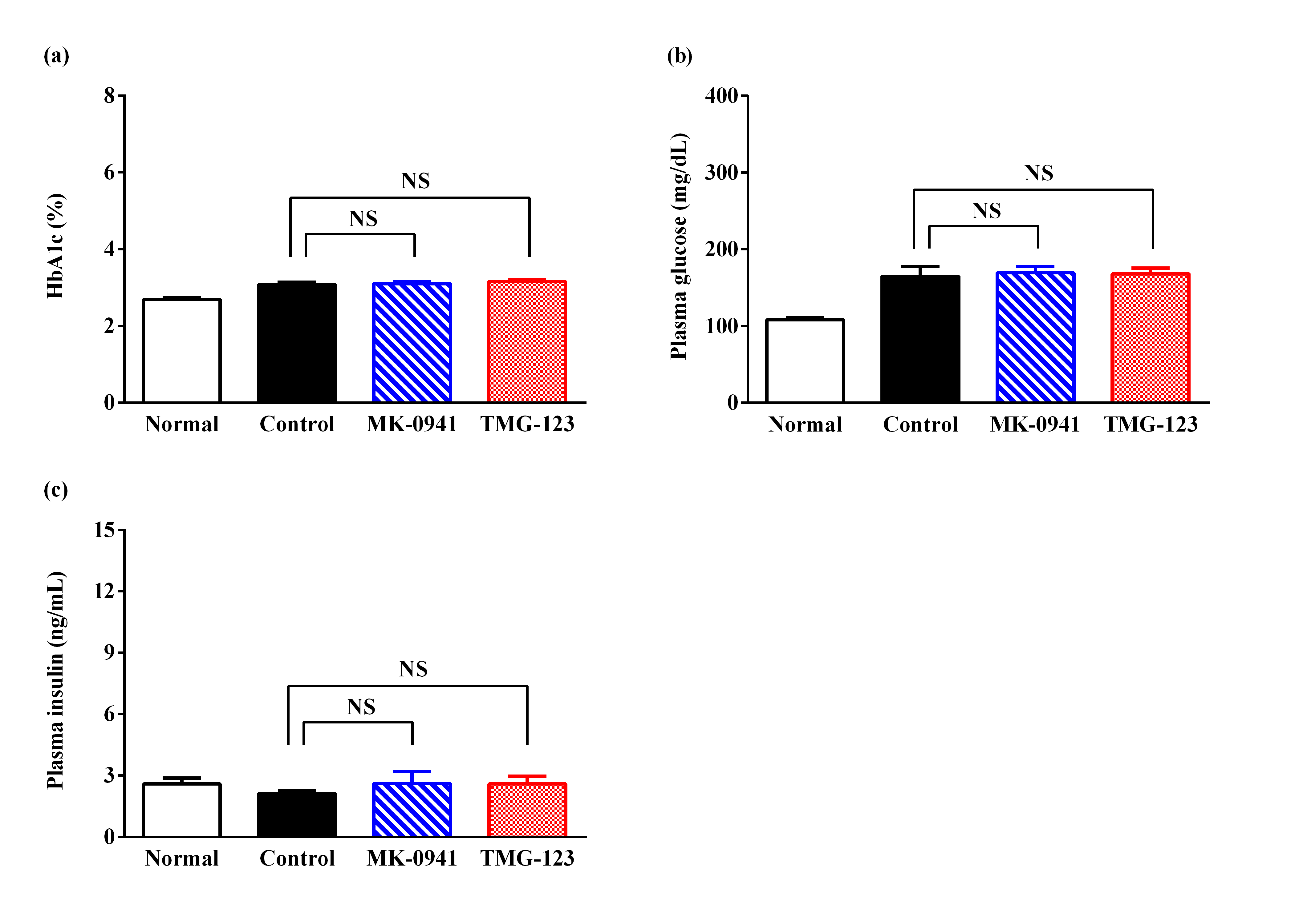
**

**S1 Fig. HbA1c level, plasma glucose, and insulin levels before treatment with GK activators**

(a) HbA1c level, (b) plasma glucose level, and (c) plasma insulin levels before treatment with GK activators (at baseline) in the chronic treatment study. Normal; Wistar rats, Control; Goto-Kakizaki rats fed normal diet in the treatment period, n=7-8. NS = not significant.
