# Supplementary material for: Disruptions in hepatic glucose metabolism are involved in the diminished efficacy after chronic treatment with glucokinase activator
Source: PLoS One. 2022 Mar 21;17(3):e0265761. doi: 10.1371/journal.pone.0265761 (PMC8936481; doi:10.1371/journal.pone.0265761)
Supplement: S2 Fig — Plasma glucagon levels at week 20 of the chronic treatment study. Normal; Wistar rats, Control; Goto-Kakizaki rats fed normal diet, n = 7–8. NS = not significant. (DOCX) [file pone.0265761.s002.docx]

**
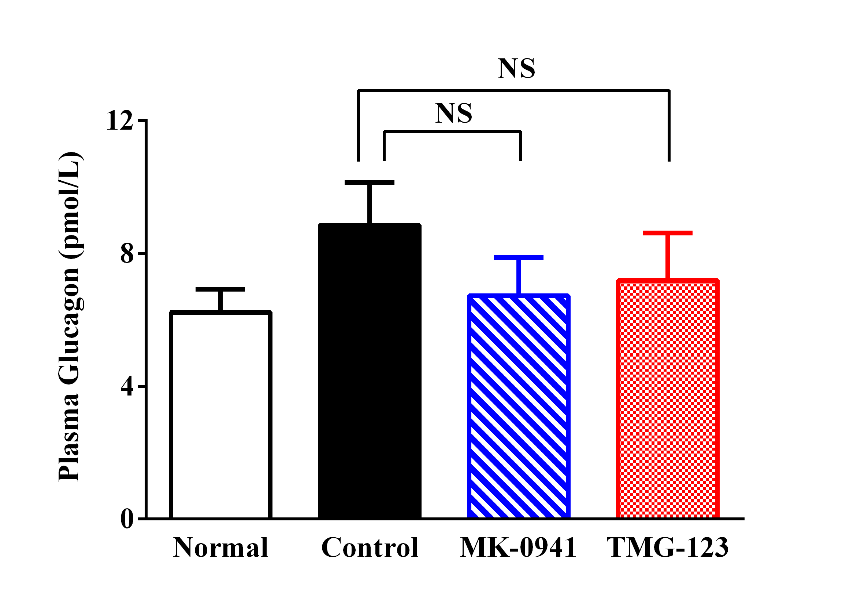
**

**S2 Fig. Plasma glucagon levels at 20-week treatment with GK activators**

Plasma glucagon levels at week 20 of the chronic treatment study. Normal; Wistar rats, Control; Goto-Kakizaki rats fed normal diet, n=7-8. NS = not significant.
